# Trade-offs in muscle physiology in selectively bred high runner mice

**DOI:** 10.1242/jeb.244083

**Published:** 2022-12-09

**Authors:** Alberto A. Castro, Theodore Garland, Saad Ahmed, Natalie C. Holt

**Affiliations:** Department of Evolution, Ecology, and Organismal Biology, University of California, Riverside, Riverside, CA 92521, USA

**Keywords:** Artificial selection, Endurance, Force–velocity, Locomotion, Muscle physiology, Trade-offs

## Abstract

A trade-off between locomotor speed and endurance occurs in various taxa, and is thought to be underpinned by a muscle-level trade-off. Among four replicate high runner (HR) lines of mice, selectively bred for voluntary wheel-running behavior, a negative correlation between average running speed and time spent running has evolved. We hypothesize that this trade-off is due to changes in muscle physiology. We studied the HR lines at generation 90, at which time one line (L3) is fixed for the mini-muscle phenotype, another is polymorphic (L6) and the others (L7, L8) lack mini-muscle individuals. We used *in situ* preparations to quantify the contractile properties of the triceps surae muscle complex. Maximal shortening velocity varied significantly, being lowest in mini-muscle mice (L3 mini=25.2 mm s^−1^, L6 mini=25.5 mm s^−1^), highest in normal-muscle mice L6 and L8 (40.4 and 50.3 mm s^−1^, respectively) and intermediate in normal-muscle L7 mice (37.2 mm s^−1^). Endurance, measured both as the slope of the decline in force and the proportion of initial force that could be sustained, also varied significantly. The slope was shallowest in mini-muscle mice (L3 mini=−0.00348, L6 mini=−0.00238), steepest in lines L6 and L8 (−0.01676 and −0.01853), and intermediate in L7 (−0.01145). Normalized sustained force was highest in mini-muscle mice (L3 mini=0.98, L6 mini=0.92) and lowest in L8 (0.36). There were significant, negative correlations between velocity and endurance metrics, indicating a muscle-level trade-off. However, this muscle-level trade-off does not seem to underpin the organismal-level speed and endurance trade-off previously reported as the ordering of the lines is reversed: the lines that run the fastest for the least time have the lowest muscle complex velocity and highest endurance.

## Teaser

**Summary:** There is a muscle-level trade-off between speed and endurance across replicated lines of mice experimentally selected for high levels of voluntary wheel running. However, this trade-off does not appear to underpin a previously reported organismal-level trade-off.

## INTRODUCTION

Trade-offs, limits to adaptation and multiple solutions have long been held as cornerstones in evolutionary biology, and in many sub-fields of organismal biology (Garland and Carter, 1994; Ackerly et al., 2000; Martin et al., 2015; Agrawal, 2020). Multiple types of trade-offs have been recognized (Cohen et al., 2020; Mauro and Ghalambor, 2020; Garland et al., 2022). Perhaps the most common type involves allocation constraints. For example, if the energy available to an organism is limited, then spending more on one function (e.g. disease resistance) means less is available for other functions (e.g. reproduction). A different type of trade-off occurs when features that enhance performance of one task decrease performance of another (Garland et al., 2022). Such functional conflicts are apparent in bone and muscle biomechanics, for example, the relative lengths of in-levers and out-levers in the skeletal system (Santana, 2016), and force–velocity trade-offs in muscle (Herrel et al., 2009; Schaeffer and Lindstedt, 2013).

In the locomotor system, the most commonly studied potential trade-off at the level of organismal performance is the sometimes-negative relationship between speed and endurance. For example, among 12 species of closely related lacertid lizards, speed and endurance capabilities are negatively related after accounting for variation in body size (Vanhooydonck et al., 2001). However, this trade-off is not apparent among species of phrynosomatid lizards (Albuquerque et al., 2015; see also Toro et al., 2004; Goodman et al., 2007). Many studies have also tested for trade-offs at the level of variation among individuals. For example, statistically significant trade-offs were detected between speed-related and endurance-related events in a study of 1369 elite human athletes participating in heptathlon and decathlon events (Careau and Wilson, 2017), and between terrestrial exertion capacity and aquatic burst performance in male tropical clawed frogs (*Xenopus tropicalis*) (Herrel and Bonneaud, 2012). When present, the organismal-level trade-off between speed and endurance is thought to be underpinned by a muscle-level trade off, presumably caused by the co-variation of myosin isoform expression and oxidative capacities across muscle fibers (e.g. see Garland, 1988).

Mammalian muscle fiber types vary along a continuum of contractile and metabolic properties (for a review see Schiaffino and Reggiani, 2011). At one end of the spectrum, Type I fibers contract slowly, use oxidative metabolism, have low power outputs, and are fatigue resistant. At the other end, Type IIb fibers contract rapidly, use glycolysis, have high power outputs, and fatigue rapidly. Type IIa fibers are intermediate, being fast-twitch, more fatigue resistant than Type IIb fibers, and using both oxidative and glycolytic metabolisms (Komi, 1984; Gleeson and Harrison, 1988; Rome et al., 1988; Esbjörnsson et al., 1993; Schiaffino and Reggiani, 2011). Muscle fiber type variation has clear links with locomotor diversity. For instance, the predominance of Type I fibers in the forelimb muscles of slow-moving sloths (Spainhower et al., 2018) contrasts with the predominance of Type IIb fibers in the hindlimb muscles of fast-sprinting cheetahs (Williams et al., 1997). The variation in locomotor performance among lizard species also seems to relate to variation in muscle fiber types (Bonine et al., 2005; Vanhooydonck et al., 2014; Albuquerque et al., 2015; Scales and Butler, 2016).

Selection experiments and experimental evolutionary approaches (Garland and Rose, 2009) present unique opportunities to study mechanisms underlying trade-offs (discussed in Garland et al., 2022). In the present study, we use four replicate lines (lab designated as lines 3, 6, 7 and 8, henceforth referred to as L3, L6, L7 and L8) of high runner (HR) mice to explore the muscular basis of organismal-level trade-offs between speed and endurance. These HR mice have been selectively bred for 90 generations based on the average number of wheel revolutions on days 5 and 6 of wheel access when young adults (Swallow et al., 1998). The HR lines evolved rapidly and reached selection limits after ∼17–27 generations (Careau et al., 2013), at which point mice from all four HR lines run approximately three-fold more than mice in the four non-selected control lines. HR mice also have increased endurance and maximal aerobic capacity (*V̇*_O_2_,max_) during forced treadmill exercise, larger hearts and larger brains, among various other phenotypic and genetic differences (Garland, 2003; Meek et al., 2009; Kolb et al., 2010, 2013; Hillis et al., 2020; Castro et al., 2022).

One striking discovery in the HR selection experiment was the ‘mini-muscle’ phenotype, characterized by a 50% reduction in the mass of the triceps surae muscle complex (Garland et al., 2002), caused primarily by a dramatic reduction in Type IIb muscle fibers (Guderley et al., 2006; Talmadge et al., 2014). The gastrocnemius muscle was considerably lighter in mini-muscle individuals and *in vitro* studies of muscle properties showed some evidence of slower twitches, altered curvature of the force–velocity relationship, reduced power production and improved endurance in this muscle (Syme et al., 2005). In contrast, the soleus muscle was 30% larger in mini-muscle mice, and its contractile properties were largely unaltered, other than the observation of some faster twitch properties in one of the mini-muscle groups (Syme et al., 2005). One of the HR lines (L3) became fixed for the mini-muscle phenotype sometime between generations 22 and 36 (Garland et al., 2002; Syme et al., 2005), while another line (L6) remains polymorphic after 95 generations (Hiramatsu et al., 2017; Cadney et al., 2021).

Across all four replicate HR lines, but not across the four control lines, Garland et al. (2011) reported a significant negative correlation between average running speed (wheel revolutions per minute) and time spent running (minutes of wheel running per day) at generation 43. In the base population, these two traits were positively correlated both phenotypically and genetically (Swallow et al., 1998), and we might expect that the evolution of an organismal speed and endurance trade-off could be related to evolved changes in lower-level traits, specifically in skeletal muscle. Therefore, the purpose of the present study was to examine muscle contractile properties to determine whether a muscle-level trade-off underlies the negative relationship between the duration of daily running and the average running speed that has evolved across the replicate HR lines.

We quantified the speed and endurance properties of an important locomotor muscle group in mice, the triceps surae complex, which contains the fast medial gastrocnemius, lateral gastrocnemius and plantaris muscles, along with the slow soleus muscle (Zhan et al., 1999; Houle-Leroy et al., 2003; Guderley et al., 2006; McGillivray et al., 2009; Schaeffer and Lindstedt, 2013; Talmadge et al., 2014). We studied this entire muscle complex *in situ*, as opposed to studying individual muscles *in vitro*, as we believe this provides the best assessment of muscle speed and endurance in the context of locomotion. In future studies, it would also be of interest to examine the contractile properties of individual muscles.

In *in situ* preparations, the muscle complex remains connected to a functioning circulatory system. This avoids the complication of diffusion limitations (Barclay, 2005) during *in vitro* endurance tests, therefore allowing for more physiologically realistic measurements of muscle endurance. In addition to retaining a connection to the circulatory system, *in situ* study of the triceps surae muscle complex allows for simultaneous activation of all muscles within this complex, and determination of its emergent contractile properties. This simultaneous activation ignores physiological recruitment (Liddell and Sherrington, 1925; Henneman, 1957; Morris and Askew, 2010; Holt et al., 2014), such as the increase in total activation of the triceps surae complex, and particularly that of faster fibers, with increasing speed and incline in running rats (Hodson-Tole and Wakeling, 2008). However, this phenomenon is also ignored in *in vitro* preparations in which muscles are typically maximally activated despite the variation in activation that occurs within a single muscle across locomotor conditions (Hodson-Tole and Wakeling, 2008; Morris and Askew, 2010). Perhaps more importantly than the somewhat unrealistic activation patterns, this approach limits our ability to attribute aspects of performance to individual muscles. However, locomotion is not powered by individual muscles, but rather driven by torques around joints produced by synergistic muscles, such as the triceps surae complex. Hence, the emergent properties of this muscle complex seem the most relevant to locomotion. This is particularly important in the HR mice, as a previous comparison of just a subset of HR lines showed that the mini-muscle phenotype has different effects on the various muscles within the triceps surae muscle complex (Syme et al., 2005). Hence, examination of any single muscle would likely not be reflective of the cumulative effects of selection.

This study used an *in situ* triceps surae complex preparation to determine the isometric twitch times and isotonic force–velocity properties as metrics of the speed of the muscle complex, and the changes in force over hundreds of isometric tetanic contractions as metrics of the endurance of the muscle complex. Although not entirely representative of muscle function during locomotion, these metrics were chosen as they provide good estimates of the bounds of muscle speed and endurance, and can be reliably measured in a multiple muscle preparation.

Based on the assumption that muscle properties at least partly underpin the organismal-level trade-off between speed and endurance, we hypothesized that muscle complex speed and endurance metrics would trade off in HR lines in a way that parallels the documented organismal variation in wheel-running speed and duration (Garland et al., 2011). At the organismal level, L8 mice ran for the longest and had the slowest mean wheel-running speeds (Garland et al., 2011), whereas L3 mice had the fastest mean speeds on wheels and ran for the shortest total duration. Hence, we hypothesized that muscle complexes from L8 mice would have the highest endurance and the lowest velocity, and muscle complexes from L3 mice would be the opposite. However, these predictions seem to be at odds with the findings of Syme et al. (2005) mentioned previously, who found L3 and L6 mini-muscle mice to generally have slower, more fatigue-resistant medial gastrocnemius muscles, with only marginal evidence for faster soleus muscles in L6 mini-muscle mice.

In contrast to Syme et al. (2005), we compared muscle properties across all four replicate lines of HR mice, and examined the speed and endurance properties of the entire triceps surae complex. This allowed us to compare any trade-offs in muscle complex properties with the organismal-level trade-offs reported across all HR lines ∼10 generations after Syme et al. (2005). Moreover, we used the knowledge gained from this prior study of a subset of HR lines (Syme et al., 2005), namely that the properties of individual muscles vary differently across lines, to inform the design of the present study in which we examined the emergent properties of the entire triceps surae complex. We believe this provides novel insight into the muscle-level determinants of organismal performance, with particular reference to the role of the trade-off between speed and endurance in the evolution of locomotor performance.

## MATERIALS AND METHODS

### The high runner mouse model

Mice from the four HR lines bred for voluntary wheel running during 6 days of wheel access as young adults were compared with four non-selected control lines (Swallow et al., 1998). Briefly, the founding population was 224 laboratory house mice (*Mus musculus domesticus* Schwarz and Schwarz 1943) of the outbred, genetically variable Hsd:ICR strain (Harlan-Sprague-Dawley, Indianapolis, IN, USA). Mice were randomly bred for two generations and then separated into eight closed lines, which consist of 10 breeding pairs each generation. During the routine selection protocol, mice were weaned at 21 days of age and housed in groups of four individuals of the same sex until ∼6–8 weeks of age. Mice were then housed individually in cages attached to computer-monitored wheels (1.12 m circumference, 35.7 cm diameter, and 10 cm wide wire-mesh running surface) with a recording sensor that counts wheel revolutions in 1-min intervals over 6 days of wheel access (Swallow et al., 1998; Careau et al., 2013; Hiramatsu, 2017). In the HR lines, the highest-running male and female from each family were chosen as breeders. The selection criterion was total wheel revolutions on days 5 and 6 to avoid potential effects of neophobia during the initial exposure to wheels. Sibling mating is not allowed. Mice were kept at room temperatures of approximately 22°C, with *ad libitum* access to food and water and a 12 h:12 h light:dark photoperiod.

### Study animals

To examine whether trade-offs in muscle properties underlie the trade-off between average running speed and duration that has evolved among the HR lines (Garland et al., 2011), we studied all four of the HR lines (L3, L6, L7 and L8). Female mice (*N*=31) from generation 90 of the selection experiment were housed four per cage beginning at weaning. We chose HR females because they generally run greater daily distances, at higher average speeds, than HR males (Garland et al., 2011), thus making it more likely that muscle-based trade-offs might be relevant.

As noted in the Introduction, the ‘mini-muscle’ phenotype presently occurs in a subset of the HR mice. In our sample of 31 mice (not all of which had data for all traits), the number of mini-muscle individuals was 6 of 6 in L3 and 5 of 11 in L6. The presence of the mini-muscle phenotype means that rather than four lines, we instead have five total groups: L3 mini (*N*=6), L6 mini (*N*=5), L6 (*N*=6), L7 (*N*=8) and L8 (*N*=6). Based on a previous study (Syme et al., 2005), we expected that these sample sizes would provide sufficient power to demonstrate any differences between groups.

All mice were housed at room temperature with food and water *ad libitum*. All experiments were approved by the University of California, Riverside, Institutional Animal Care and Use Committee. Given the time it takes to perform surgical experiments on individuals from all four replicate lines of HR mice, the use of mice from a single generation, and because of breeding constraints owing to the nature of the HR mouse selection experiment (see Swallow et al., 1998), there was some necessary variation in age. Mice ranged from 46 to 107 days old. To account for this variation, age was included as a covariate in all analyses.

### Surgical procedure

The twitch, tetanic, force–velocity and endurance properties of the left triceps surae muscle complex were determined *in situ*. Mice were anesthetized (SomnoSuite Low-flow Anesthesia System, Kent Scientific, Torrington, CT, USA) and maintained at 1.5–5% isoflurane anesthesia. The depth of anesthesia was continually monitored, and the dosage adjusted to maintain a sufficient depth. Body temperature was monitored using a thermometer inserted into the rectum, and maintained throughout surgery via an integrated system that continuously adjusted the temperature of the heat pad placed under the animal (RightTemp System, Kent Scientific). The sciatic nerve was surgically exposed, and a bipolar nerve cuff for electrical stimulation of the triceps surae complex was placed around it. Mineral oil was applied at the attachment site to keep the nerve moist, and the incision was closed. The proximal end of the femur was exposed and clamped into a custom-made stereotaxic frame. The Achilles tendon was exposed distally, Kevlar thread tied tightly around it, and the calcaneus cut. The end of the free tendon was attached to the lever arm of a servomotor (305C-LR Dual-Mode Lever System, Aurora Scientific, Aurora, ON, Canada), allowing for measurements of muscle force, length and velocity in the triceps surae complex (Ranatunga, 1984; Claflin and Faulkner, 1989; Zhan et al., 1999; Syme et al., 2005; Holt et al., 2016; Javidi et al., 2020).

### Muscle activation and data logging

All recordings and data processing were performed using data acquisition software (IgorPro 7, WaveMetrics, Lake Oswego, OR, USA). Stimulation protocols were sent to the muscle, and data logged, at a sampling frequency of 10,000 Hz using a DAQ AD board (CompactDAQ, National Instruments, Austin, TX, USA). Supramaximal square wave pulses (amplitude 1–2 mA, pulse duration 0.1 ms) were constructed (IgorPro 7, WaveMetric) and applied to the sciatic nerve (CompactDAQ, National Instruments; High-Power, Biphase Stimulator, Aurora Scientific) (Holt and Azizi, 2014). The brief pulse duration (0.1 ms) required to prevent damage to the sciatic nerve necessitated the high frequency sampling used (10,000 Hz). Single pulses were used to elicit all twitch contractions, whereas 350 ms trains of pulses delivered at 80 Hz were used to elicit all tetanic contractions. The stimulus amplitude was adjusted, and elicited twitch force determined, in every muscle complex. The lowest stimulus amplitude giving peak twitch force was used for all subsequent contractions. Pulse frequency and train duration were varied for a subset of muscle complexes, and the degree of fusion and force plateau were examined. A stimulation frequency of 80 Hz consistently produced a fused tetanic contraction in which force fluctuations owing to individual pulses were <1% of total force, and a train duration of 350 ms gave a clear force plateau during isometric tetani. Visual inspection of subsequent muscle complexes confirmed that these stimulation parameters produced these effects across all individuals.

### Muscle isometric properties

Isometric twitch and tetanic properties of the triceps surae complex were determined. Initially, a series of twitches were performed at a range of lengths. The length that yielded peak twitch force was determined, and defined as optimum length (*L*_0_). All subsequent contractions were performed at this length, and all forces were corrected for passive force at this length.

A subsequent twitch contraction was performed at optimum length. Peak twitch force was determined and time series data were used to calculate the time from onset of activation to peak twitch force (TP_tw_), and the time from peak twitch force to 50% relaxation (TR_50_) (Marsh and Bennett, 1985, 1986; Bennett et al., 1989; Askew and Marsh, 1997; Syme et al., 2005; Nguyen et al., 2020) (Table 1).

**Table 1.**
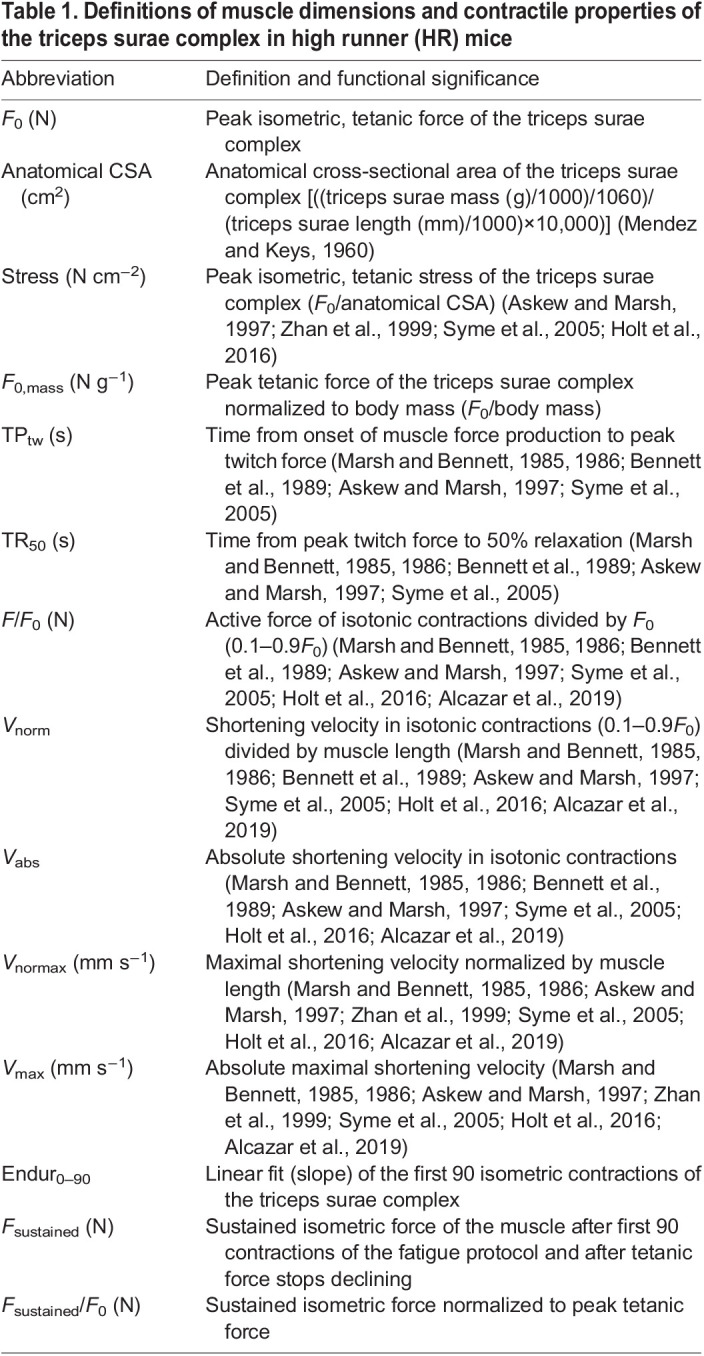
Definitions of muscle dimensions and contractile properties of the triceps surae complex in high runner (HR) mice

Next, an isometric tetanic contraction was performed to determine peak isometric tetanic force (*F*_0_) (Table 1). Control isometric tetanic contractions were repeated at regular intervals to monitor muscle complex performance (Holt and Azizi, 2014; Holt et al., 2016). It was pre-established that if force had dropped below 90% of its initial value by the first control isometric tetani (∼fifth tetanic contraction), the experiment would be terminated. Large drops in force between the first and ∼fifth tetanic contractions resulted in termination of the experiment in 11 mice. We found these HR mice to be particularly sensitive to the effects of both anesthesia and nerve stimulation, hence requiring the exclusion of many mice. However, we believe that the termination criteria used here allow for minimization of these effects without biasing our sample towards more fatigue-resistant muscle complexes.

Following muscle experiments, muscle complexes were weighed, cross-sectional area (CSA) was calculated (see below) and peak isometric stress was calculated as peak isometric tetanic force divided by CSA. Peak tetanic force (*F*_0_) was also normalized to body mass (*F*_0,mass_) to assess the capacity of the muscle complex to support body weight during locomotion (Table 1).

### Muscle force–velocity properties and curve fitting

To determine the relationship between muscle complex force and velocity, isotonic tetanic contractions in which the muscle was allowed to shorten were performed at a range of relative forces (0.1–0.9*F*_0_). Peak shortening velocity was determined at each of these force levels (Fig. S1) and force–velocity curves were constructed (Marsh and Bennett, 1985, 1986; Bennett et al., 1989; Askew and Marsh, 1997; Zhan et al., 1999; Syme et al., 2005; Holt et al., 2016; Alcazar et al., 2019; Javidi et al., 2020). For each muscle complex, we performed 13 total contractions, included isotonic shortening contractions and isometric controls, during the force–velocity protocol. This consistency ensured that muscle complexes from all individuals were in the same state at the beginning of the endurance protocol.

The force–velocity data were normalized. Active forces in isotonic contractions were divided by peak isometric tetanic force to determine relative force (*F*/*F*_0_). Following muscle experiments, the length of the muscle complex was measured and absolute shortening velocities (*V*_abs_) were divided by this length to calculate normalized shortening velocity (*V*_norm_) (Table 1). After plotting the force–velocity points for individual mice, we fitted force–velocity curves using multiple equations. We initially chose not to rely on a single force–velocity curve fit as none of the commonly used fits have a mechanistic basis, and the force–velocity curves characterized here were relatively linear compared with previously observed curves (see Marsh and Bennett, 1986; Alcazar et al., 2019). We fitted force–velocity data using the Hill rectangular-hyperbola equation: (*P*+*a*)(*v*+*b*)=*b*(*P*_0_+*a*) (Hill, 1938); the Marsh–Bennett hyperbolic linear equation: *V*=*B*(l−*F*/*F*_0_)/(*A*+*F*/*F*_0_)+*C*(1−*F*/*F*_0_) (Marsh and Bennett, 1986; Askew and Marsh, 1997); and a second-order polynomial: *V*=*Ax*^2^+*Bx*+*C*. Maximal shortening velocity values were determined (Table 1) for the three fits for all mice (Fig. S3), and curves were visually rendered to check for poor fits. Force–velocity data for four mice were excluded owing to poor curve fits. The force–velocity relationships in these individuals showed negative quadratic fits, whereas the rest of the individuals all had positive quadratic terms.

### Muscle endurance properties

The force-generating capacity of the triceps surae muscle complex over repeated isometric tetanic contractions was used to assess muscle endurance (Renaud and Kong, 1991; Zhan et al., 1999; James et al., 2004; Syme et al., 2005). The use of an *in situ* muscle preparation eliminated the effects of the central nervous system while maintaining blood supply and, therefore, provided an assessment of the muscular basis of endurance. The endurance protocol consisted of a standard procedure of repeated isometric tetanic contractions (Allen et al., 2008) elicited using the same stimulation parameters as previous isometric tetanic contractions. One contraction was performed every 5 s until force dropped below 50% of its initial value, or for a maximum of 500 contractions. However, owing to the high sampling frequency required, these contractions had to be performed in 100-contraction bouts. At the end of each bout the data were saved, and a new bout immediately started.

Peak force in each individual contraction was calculated and plotted against contraction number (∼200–500 contractions) (Fig. S2). Endurance (Endur_0–90_) was quantified as the linear fit (slope) of the decline in force over the first 90 tetanic contractions (Table 1). It was not our intention that this linear descriptor would provide a precise fit to the data. Instead, it provided a comprehensive and comparable way to capture the duration for which initial force can be maintained, the rate of a decline in force, and the point at which force could be sustained, thus allowing comparison across the HR lines. After the first 90 contractions, we quantified the average force that was sustained (*F*_sustained_) over a series of tetanic contractions without a decrease in force (Table 1). We made sure to quantify *F*_sustained_ over areas in which force traces were consistently flat and without any peaks (see Results). This sustained force was normalized to peak isometric force measured at the beginning of the experiment (*F*_sustained_/*F*_0_) to quantify the decline in active force given the different levels of initial force across the lines.

### Dissections and muscle dimensions

Once the endurance contraction protocol was completed, an overdose of isoflurane anesthesia was administered. The lengths of the Achilles tendon, triceps surae muscle complex (length from the knee joint to the origin of the common Achilles tendon) and muscle–tendon unit were measured to the nearest 0.1 mm with digital calipers while the mouse was still in the stereotaxic frame and the muscle was held at *L*_0_. Mice were then removed from the frame, decapitated and weighed. The triceps surae complex was dissected free and weighed to the nearest 0.0001 g.

Muscle complex anatomical cross-sectional area (anatomical CSA) (not accounting for pennation angle or fiber length) was determined from muscle complex mass and length assuming a density of 1060 kg m^−3^ (Mendez and Keys, 1960) (Table 1). Subsequently, we calculated the peak tetanic stress (stress=*F*_0_/CSA) of the triceps surae muscle group (Askew and Marsh, 1997; Zhan et al., 1999; Syme et al., 2005; Holt et al., 2016) (Table 1). Muscle shortening velocities were normalized to muscle complex length.

### Statistical analysis

#### Isometric contractile properties

To compare the five groups (four HR lines, with L6 divided into those with and without the mini-muscle phenotype), we used the MIXED Procedure in SAS (SAS Institute, Cary, NC, USA) to apply analysis of covariance models with age as the covariate. This inclusion of age is necessary because of the large range of ages included in this study (46–107 days old). Analyses of muscle dimensions (except for variables that were normalized) also included body mass as a covariate. We calculated an *a priori* contrast comparing L3 mini and L6 mini with L6, L7 and L8. For *post hoc* comparisons within the mini- and normal-muscled groups, we examined differences of least squares means from SAS Procedure MIXED, with adjustment for multiple comparisons. Specifically, we employed Scheffe's procedure because this is the most conservative multiple-range comparison for unequal sample sizes. No data were excluded from isometric contractions, except for *F*_0,mass_, for which one low outlier was removed from the analyses. In the endurance protocol we were missing some values of sustained force because during early experiments, the duration of the endurance protocol required to achieve a reliable value of sustained force was not clear and in a small number of cases, the protocol was stopped prematurely.

#### Force–velocity repeated measures

Multiple force–velocity points were obtained for each individual mouse, so we used repeated-measures models in SAS Procedure MIXED to test for effects of group on both absolute shortening velocity (*V*_abs_) and normalized velocity (*V*_norm_). Covariates were age, relative force (*F*/*F*_0_) and *z*-transformed relative force squared [*Z*(*F*/*F*_0_)^2^; orthogonal polynomial used to describe the curvature of the relationship]. Individual was treated as a random effect nested within line. Furthermore, we included the interaction between force (*F*/*F*_0_) and group (*F*/*F*_0_×group) to test for differences in slopes. Initially, we also included the interaction between *Z*(*F*/*F*_0_)^2^ and group [*Z*(*F*/*F*_0_)^2^×group] to test for differences in curvature, but this interaction was not significant, so it was removed from the final model we present.

Least-square means generated from the repeated-measures analyses were estimated at *F*/*F*_0_=0 to estimate maximal shortening velocity (mm s^−1^) values from the second-degree polynomials for both absolute (*V*_max_) and normalized velocity (*V*_normax_).

#### Correlations of muscle traits

To examine covariation of muscle complex performance metrics among the five groups, we examined bivariate scatterplots and calculated Pearson pairwise correlation coefficients for *V*_normax_, Endur_0–90_, stress, TP_tw_, TR_50_ and *F*_sustained_/*F*_0_. We also attempted to calculate correlations while accounting for within-group variation, as indicated by the standard errors, using procedures outlined in Ives et al. (2007), but the data set was too small to achieve reasonable estimates.

## RESULTS

**Fig. 1.**
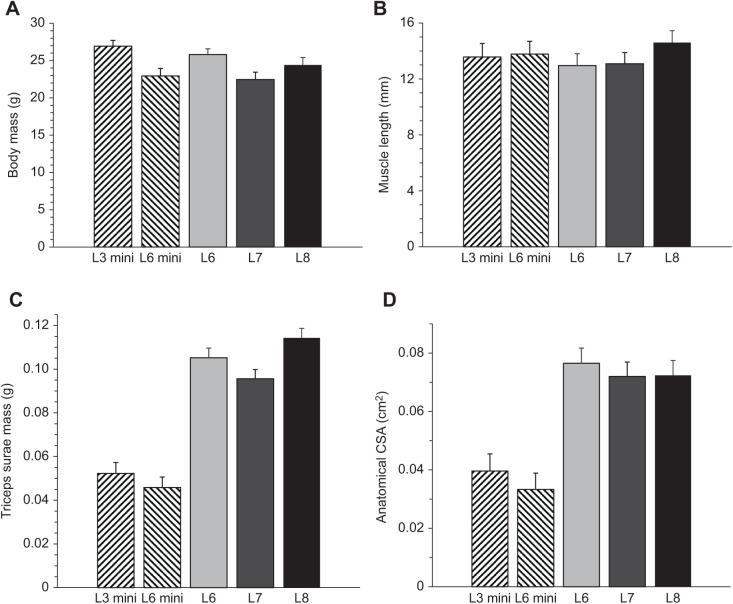
**Body mass and muscle dimensions for all groups of mice.** (A) Least square means and standard errors of body mass for each line (L3 mini, L6 mini, L6, L7 and L8). Age was positively associated with body size and both L6 mini and L7 mice were significantly lighter when compared with the other lines. Type 3 tests of fixed effects: group, *F*_4.25_=6.16, *P*=0.0014; age, *F*_1,25_=12.22, *P*=0.0018. (B) Least square means and standard errors of triceps surae muscle length for each line. Neither age nor body mass was associated with muscle length, and the lines did not differ significantly. Type 3 tests of fixed effects: group, *F*_4,24_=0.69, *P*=0.6064; body mass, *F*_1,24_=0.65, *P*=0.4284; age, *F*_1,24_=0.20, *P*=0.6553. (C) Least square means and standard errors of triceps surae muscle mass for each line. Triceps surae muscle mass was positively associated with body mass, and mini-muscle mice (L3 mini and L6 mini) had significantly lighter muscles when compared with the other lines (L6, L7 and L8), with L7 having intermediate values. Type 3 tests of fixed effects: group, *F*_4,24_=50.67, *P*<0.0001; body mass, *F*_1,24_=2.91, *P*=0.1007; age, *F*_1,24_=0.00, *P*=0.9735. (D) Least square means and standard errors of anatomical cross-sectional area (CSA) for each line. Mini-muscle mice (L3 mini and L6 mini) had significantly lower anatomical CSA values when compared with the other lines. Type 3 tests of fixed effects: group, *F*_4,24_=16.72, *P*<0.0001; body mass, *F*_1,24_=2.01, *P*=0.1696; age, *F*_1,24_=0.88, *P*=0.3585. L3 mini *N*=6, L6 mini *N*=5, L6 *N*=6, L7 *N*=8 and L8 *N*=6 for all traits presented in this figure.

**Fig. 2.**
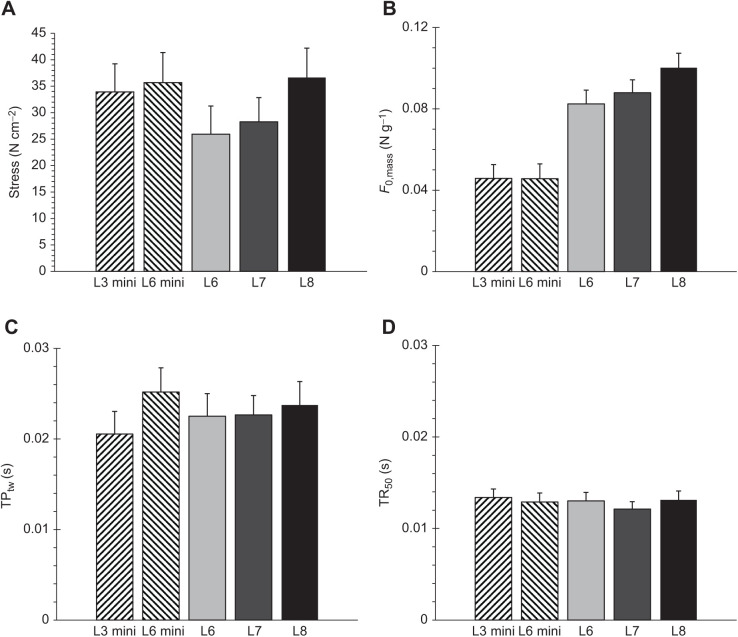
**Isometric contractile properties for all groups of mice.** (A) Least square means and standard errors of stress for each line (L3 mini, L6 mini, L6, L7 and L8). Neither age nor body mass was associated with stress and the lines did not differ significantly. Type 3 tests of fixed effects: group, *F*_4,25_=0.86, *P*=0.5007; age, *F*_1,25_=0.04, *P*=0.8386. (B) Least square means and standard errors of *F*_0,mass_ for each line. Mini-muscle mice (L3 mini and L6 mini) had significantly lower *F*_0,mass_ values when compared with the other lines (L6, L7 and L8). Type 3 tests of fixed effects: group, *F*_4,24_=11.74, *P*<0.0001; age, *F*_1,24_=1.19, *P*=0.2868. (C) Least square means and standard errors of TP_tw_ for each line. Neither age nor body mass was associated with TP_tw_, and the lines did not differ significantly. Type 3 tests of fixed effects: group, *F*_4,25_=0.45, *P*=0.7687; age, *F*_1,25_=0.15, *P*=0.6987. (D) Least square means and standard errors of TR_50_ for each line. Neither age nor body mass was associated with TR_50_, and the lines did not differ significantly. Type 3 tests of fixed effects: group, *F*_4,25_=0.32, *P*=0.8626; age, *F*_1,25_=0.29, *P*=0.5919. L3 mini *N*=6, L6 mini *N*=5, L6 *N*=6, L7 *N*=8 and L8 *N*=6 for all traits presented in this figure with the exception of *F*_0,mass_, where L7 *N*=7.

**Fig. 3.**
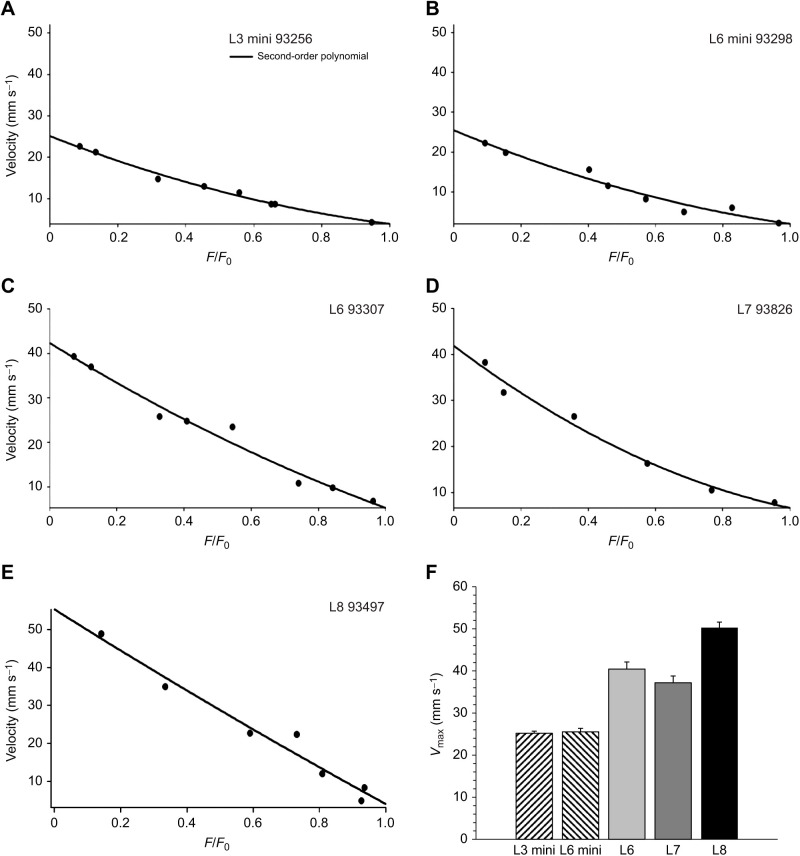
**Sample and summary force–velocity data for all groups of mice.** (A) Representative force–velocity trace for L3 mini, with *F*/*F*_0_ on the *x*-axis and absolute shortening velocity on the *y*-axis. The force–velocity points were curve-fitted using the second-order polynomials and maximal shortening velocity mm s^−1^ estimates using this fit are visually rendered. (B) Representative force–velocity trace for L6 mini. (C) Representative force–velocity trace for L6. (D) Representative force–velocity trace for L7. (E) Representative force–velocity trace for L8. (F) Least square means and standard errors of *V*_max_ *F*_0,mass_ for each line based on second-order polynomials. *V*_max_ was positively associated with age, and mini-muscle mice (L3 mini and L6 mini) had significantly lower *V*_max_ values when compared with the other lines (L6, L7 and L8). Type 3 tests of fixed effects: group, *F*_4,180_=102.44, *P*<0.0001; age, *F*_1,180_=85.85, *P*<0.0001. Of the normal-muscle lines, L8 had the highest *V*_max_ value and L6 and L7 were intermediate. The repeated-measures design of the force–velocity experiment meant there were a total of 192 total data points. Of these there were 44 data points from six individuals in L3 mini, 33 data points from five individuals in L6 mini, 30 data points from four individuals in L6, 53 data points from seven individuals in L7, and 32 data points from five individuals in L8.

**Fig. 4.**
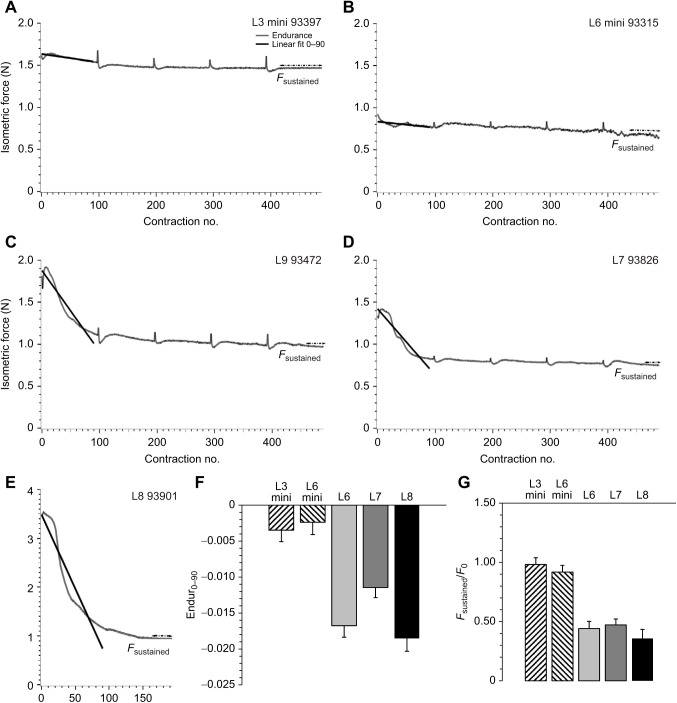
**Sample and summary endurance data for all groups of mice.** (A) Representative endurance trace wave profile for L3 mini, with contraction number on the *x*-axis and isometric force on the *y*-axis. The linear fit (Endur_0–90_) of the decline in force over the first 90 tetanic contractions and the average sustained force (*F*_sustained_) are visually rendered. (B) Representative endurance trace wave profile for L6 mini. (C) Representative endurance trace wave profile for L6. (D) Representative endurance trace wave profile for L7. (E) Representative endurance trace wave profile for L8. L8 mice all fatigued within the first 200 contractions. (F) Least square means and standard errors of Endur_0–90_ for each line. L3 mini *N*=6, L6 mini *N*=5, L6 *N*=6, L7 *N*=8 and L8 *N*=5. Endur_0–90_ was positively associated with age, and mini-muscle mice (L3 mini and L6 mini) had significantly lower Endur_0–90_ values when compared with the other lines (L6, L7 and L8), with L7 having intermediate Endur_0–90_ values. Type 3 tests of fixed effects: group, *F*_4,24_=19.52, *P*<0.0001; age *F*_1,234_=12.94, *P=*0.0014. (G) Least square means and standard errors of *F*_sustained_/*F*_0_ for each line. L3 mini *N*=6, L6 mini *N*=5, L6 *N*=5, L7 *N*=6 and L8 *N*=3 mini-muscle mice (L3 mini and L6 mini) had significantly lower *F*_sustained_/*F*_0_ values when compared with the other lines. Type 3 tests of fixed effects: group, *F*_4,19_=22.17, *P*<0.0001; age, *F*_1,19_=0.33, *P*=0.5711.

**Fig. 5.**
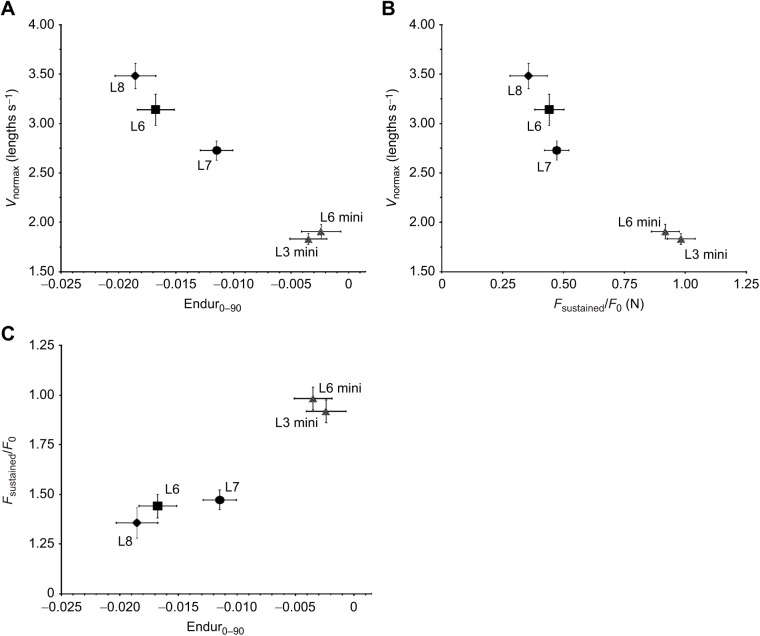
**Correlations between velocity and endurance metrics across all groups of mice.** (A) Scatterplot of least squares means and standard errors for *V*_normax_ (normalized maximal shortening velocity) and Endur_0–90_ (linear slope of the first 90 contractions). *V*_normax_ and Endur_0–90_ have a negative relationship. Mini-muscle mice (L3 mini and L6 mini) have the highest endurance (Endur_0–90_) but slowest muscles (*V*_normax_), L6 and L8 have the lowest endurance but fastest muscles, and L7 is intermediate. (B) Scatterplot of least squares means and standard errors for *V*_normax_ and *F*_sustained_/*F*_0_ (normalized force that can be sustained). *V*_normax_ and *F*_sustained_/*F*_0_ have a negative relationship. Mini-muscle mice (L3 mini and L6 mini) have the highest sustained force (*F*_sustained_/*F*_0_) but slowest muscles (*V*_normax_), L8 has the lowest sustained force but the highest *V*_normax_. (C) Scatterplot of least squares means and standard errors for *F*_sustained_/*F*_0_ and Endur_0–90_. *F*_sustained_/*F*_0_ and Endur_0–90_ have a positive relationship as would be expected given that they are both metrics of muscle endurance. *N*-values are as for Figs 3 and 4.

Significance levels from ANCOVAs of body mass, muscle complex dimensions and isometric (tetanic and twitch) properties of the triceps surae complex in HR mice (using body mass and age as a covariate when appropriate) are shown in Figs 1 and 2. Table 2 and Fig. 3 illustrate the results of force–velocity analysis, including representative traces from all groups. Fig. 4 depicts the significance values from the endurance metrics, including representative traces. Table 3 shows the pairwise correlation for the primary muscle contractile characteristics, and Fig. 5 illustrates the significant, negative correlations between velocity and endurance metrics.

**Table 2.**
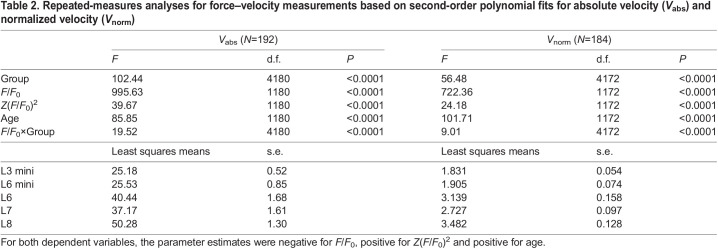
Repeated-measures analyses for force–velocity measurements based on second-order polynomial fits for absolute velocity (*V*_abs_) and normalized velocity (*V*_norm_)

**Table 3.**
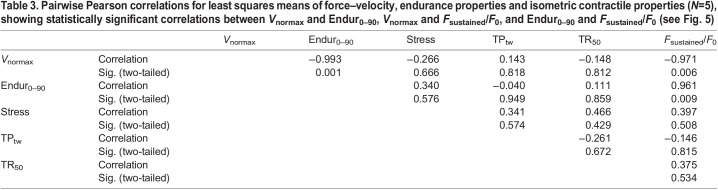
Pairwise Pearson correlations for least squares means of force–velocity, endurance properties and isometric contractile properties (*N*=5), showing statistically significant correlations between *V*_normax_ and Endur_0–90_, *V*_normax_ and *F*_sustained_/*F*_0_, and Endur_0–90_ and *F*_sustained_/*F*_0_ (see Fig. 5)

### Body size and muscle dimensions

Average body mass varied significantly among groups (*P*=0.0014) (Fig. 1A). With body mass as a covariate, muscle complex length (Fig. 1B), tendon length and muscle–tendon unit length were not significantly different among groups. As expected, relative triceps surae complex mass varied among groups (*P*≪0.0001) (Fig. 1C), with the mini-muscle mice (L3 mini and L6 mini) having significantly lighter muscle complexes (least squares means of 0.052 and 0.046 g, respectively) when compared with normal-muscled mice (L6=0.105 g, L7=0.095 g, L8=0.114 g) (*a priori* contrast *P*≪0.0001). *Post hoc* comparisons indicated no statistically significant differences between the two mini-muscle groups or among the three normal-muscle groups. The pattern for anatomical CSA was similar to that of muscle complex mass (Fig. 1D).

### Isometric properties

Isometric tetanic stress was not significantly different among groups (Fig. 2A). However, *F*_0,mass_ (peak tetanic force normalized to body mass) was significantly different among groups (*P*<0.0001) (Fig. 2B), with the main difference being that mini-muscle mice (L3 mini and L6 mini) had significantly lower values (both 0.046 N g^−1^) when compared with the other groups (0.082 N g^−1^ for L6, 0.087 N g^−1^ for L7 and 0.100 N g^−1^ for L8) (*a priori P*≪0.0001).

TP_tw_, time from onset of muscle force to peak twitch force (Table 1), ranged from an average of 0.021 s for L3 mini to 0.025 s for L6 mini, but was not significantly different among groups (Fig. 2C). TR_50_, time from peak twitch force to half relaxation (Table 1), also did not differ among groups (Fig. 2D).

### Force–velocity properties

Fig. 3 depicts force–velocity traces from a representative mouse from each group, along with the second-order polynomial curve fits (see Materials and Methods). This curve fit was deemed to provide the most reliable fit for the force–velocity points and estimation of maximal shortening velocity (*V*_max_) (Fig. 3A–E). The Hill equation forced a curve when none existed, and the Marsh–Bennett equation often generated convex shapes (Fig. S3).

For absolute velocity (*V*_abs_), the effect of group was highly significant (both *P*≪0.0001), as was the effect of relative force (*F*/*F*_0_) (both *P*≪0.0001), the *z*-transformation of force (*F*/*F*_0_) [*Z*(*F*/*F*_0_)^2^] (*P*≪0.0001), and the interaction between *F*/*F*_0_ and group (*P*≪0.0001) (Table 2). The interaction between *F*/*F*_0_ and group indicates differences in slope of the force–velocity curve among the groups. The *a priori* contrast between *V*_max_ for mini- and normal-muscled groups was highly significant (*P*≪0.0001). In addition, the *post hoc* comparisons indicated that *V*_max_ in L8 was significantly higher than L6 (*P*=0.0005) and L7 (*P*≪0.0001). Results were similar for normalized velocity (*V*_norm_) (Table 2).

### Endurance properties

Fig. 4 illustrates the endurance protocol for representative mice from each of the five groups. The slight recovery in active force every 100 contractions is due to the need to save data and restart the protocol, thus giving the muscle a slightly longer recovery time. In L3 mini and L6 mini individuals there was a minimal drop in active force over the entire endurance protocol as compared with the other three groups (e.g. Fig. 4A,B versus Fig. 4C–E). Endur_0–90_ (the slope of the decline in force over the first 90 tetanic contractions) was significantly different among groups (*P*≪0.0001), being shallowest in the mini-muscle mice (L3 mini=−0.00348, L6 mini=−0.00238), steepest in normal lines L6 and L8 (−0.01676 and −0.01853, respectively), and intermediate in L7 (−0.01145) (Fig. 4F). The *a priori* contrast between mini- and normal-muscled groups was highly significant (*P*≪0.0001). *F*_sustained_/*F*_0_ also differed among groups (*P*<0.0001), with mini-muscle groups having higher values (0.98 for L3 mini and 0.92 for L6 mini) when compared with L6 (0.44), L7 (0.47) and L8 (0.36) groups (Fig. 4G). The *a priori* contrast between mini- and normal-muscled groups was also highly significant (*P*≪0.0001).

### Pairwise Pearson's correlations

Table 3 provides correlations for the five groups least squares means for normalized maximum shortening velocity (*V*_normax_), endurance (Endur_0–90_, *F*_sustained_/*F*_0_) and isometric properties (stress, TP_tw_, TR_50_). Of the 15 correlations, the correlations between *V*_normax_ and Endur_0–90_ (*r*=−0.993), *V*_normax_ and *F*_sustained_/*F*_0_ (−0.971), and *F*_sustained_/*F*_0_ and Endur_0–90_ (0.961) were statistically significant (*P*<0.01) (Table 3, Fig. 5). Mini-muscle mice (L3 mini and L6 mini) had the highest endurance (Endur_0–90_ and *F*_sustained_/*F*_0_) but slowest muscle complexes (*V*_normax_), L6 and L8 had the lowest endurance but fastest muscle complexes, and L7 was intermediate.

## DISCUSSION

The purpose of the present study was to test whether a muscle-level trade-off underlies the negative relationship between the duration of daily wheel running and the average running speed, that was previously observed to have evolved among four replicate lines of HR mice (Garland et al., 2011). We used an *in situ* preparation of the triceps surae complex to determine muscle isometric, force–velocity and endurance properties. Although we found a negative relationship between muscle complex speed and endurance (Fig. 5A,B), indicative of a muscle-level trade-off, the ordering among lines (groups) was reversed as compared with wheel-running behavior (Garland et al., 2011).

### Muscle dimensions

The only reported difference in muscle dimensions across our HR groups was the previously reported ∼50% reduction in triceps surae muscle complex mass in mini-muscle mice when compared with normal-muscled individuals (Fig. 1C) (Garland et al., 2002; Houle-Leroy et al., 2003; Syme et al., 2005). Given that there was no statistical difference in muscle length across all groups, anatomical CSA was therefore also significantly reduced in the mini-muscle groups (Fig. 1).

### Isometric properties

Isometric tetanic stress ranged from an average of 25.9 N cm^−2^ for L6 to 36.6 N cm^−2^ for L8, but was not significantly different among groups. This calculation of stress (Table 1, Fig. 2A) was based on the anatomical CSA of the triceps surae complex. The lower value of anatomical, as opposed to physiological, CSA will lead to higher estimates of stress. Previous studies of isolated muscles from HR mice have reported values of 16.7–17.8 N cm^−2^ for the medial gastrocnemius muscle (Zhan et al., 1999), and 16.7–29.5 N cm^−2^ and 33.3–38.1 N cm^−2^ for isolated medial gastrocnemius and soleus muscles, respectively (Syme et al., 2005). Studies of isolated calf muscles (soleus and extensor digitorium longus) in CD-1 mice reported variable stress values that depended on age and fatigue (James et al., 2004; Hill et al., 2020), but were on average lower than stress values for the triceps surae complex reported here. Although our study of the entire triceps surae complex provides the most relevant measures of muscle properties as they pertain to locomotion, it also means that we cannot attribute force contributions to individual muscles, calculate physiological CSA of each of these morphological distinct muscles, nor determine stress in each individual muscle.

In addition to normalizing isometric tetanic force of the triceps surae complex to its anatomical CSA, we also normalized it to body mass to enable us to assess the capacity of this muscle group to support body weight during locomotion. Muscle force relative to body mass was significantly lower in mini-muscle mice than normal-muscle mice (Fig. 1B), which may contribute to the reduced maximal sprint speed previously observed in these groups (Dlugosz et al., 2009).

Rates of force development and relaxation were determined from isometric twitches. Time from onset of muscle complex force production to peak twitch force ranged from an average of 0.021 s for L3 mini to 0.025 s for L6 mini, but was not significantly different among groups (Fig. 2C). TR_50_ ranged from 0.012 s for L7 to 0.013 s for L8, and was also not significantly different among groups. Force rise times were a little slower than the 0.016 s measured in isolated soleus muscles from ICR outbred mice, while the half-relaxation times were slightly faster (0.023 s reported previously) (Askew and Marsh, 1997). The lack of difference between our five groups is somewhat in contrast with a previous study comparing only mini-muscle and normal-muscle groups of mice. Syme et al. (2005) showed a shorter entire twitch duration (measured at 50% of peak force) and relaxation time (measured from 90% to 10% of peak force) in the soleus from L6 mini compared with the soleus from either L3 mini or L6 normal, and a shorter relaxation time (measured from 90% to 10% of peak force) in the medial gastrocnemius muscle from L6 normal mice compared with either of the mini-muscle groups.

Faster relaxation times are not surprising given that we examined not only the slow soleus, but also the plantaris and medial and lateral gastrocnemius muscles, which are known to have a larger proportion of faster fibers (Zhan et al., 1999; Houle-Leroy et al., 2003; Guderley et al., 2006; McGillivray et al., 2009; Schaeffer and Lindstedt, 2013; Talmadge et al., 2014). The slower rate of force development, and the lack of difference across our five groups, are harder to explain. However, they may be a consequence of using the entire triceps surae complex containing multiple muscles, and significant series compliance in the Achilles tendon and aponeuroses. The use of multiple muscles within this complex may mean that any effects, such as loss of Type IIb fibers in the gastrocnemius muscles of mini-muscle mice (Guderley et al., 2006; Talmadge et al., 2014) and the slower relaxation this presumably caused (Syme et al., 2005), are obscured or counteracted by the effects of the mini-muscle phenotype on other muscles, such as the faster twitch kinetics in the soleus (Syme et al., 2005). In addition, the presence of series compliance will have slowed the time course of force generation (Hill, 1951; Mayfield et al., 2016), and may potentially have obscured any differences in rate of fiber force generation across the groups. Hence, the use of the entire triceps surae complex limits our ability to identify changes in individual muscles. However, it demonstrates that any variation in the properties of individual muscles across HR lines will likely not have affected the rate of force generation at the ankle during locomotion, and that this rate may be lower than that predicted by isolated muscle kinetics.

### Force–velocity properties

Estimated maximum shortening velocity (*V*_max_) and slope, but not curvature, of the force–velocity relationship varied across our five groups of HR mice. *V*_max_ was lowest in mini-muscle mice (L3 mini=25.2, L6 mini=25.5 mm s^−1^), highest in L8 (50.3 mm s^−1^), and intermediate in L6 and L7 (40.4 and 37.2 mm s^−1^, respectively) (Fig. 3). The values of *V*_max_ reported here are somewhat lower than have previous been documented in both isolated muscles from a subset of HR groups, ∼62–65 and ∼60–70 mm s^−1^ in isolated soleus and medial gastrocnemius muscles, respectively (Syme et al., 2005), and in other non-HR soleus muscles, where values of ∼60–65 mm s^−1^ have been reported (Asmussen and Maréchal, 1989; Maréchal and Beckers-Bleukx, 1993; Askew and Marsh, 1997). In addition, we report significant differences in *V*_max_, without any difference in the curvature of the force–velocity relationship. This is in contrast to a previous study on a subset of HR groups that showed a difference in curvature, in the absence of a difference in *V*_max_, between medial gastrocnemius muscles from mini- and normal-muscle mice (Syme et al., 2005).

The overall lower values of *V*_max_ reported here may have several explanations. It may be partially a consequence of our force–velocity curve fitting; the relatively flat force–velocity relationships measured here were fit better by a second-order polynomial than by traditional curve-fitting equations. This approach may have reduced the estimate of *V*_max_ compared with other curve-fitting methods (Fig. S3). However, it is possible that the relatively low value of *V*_max_ also reflects a shift in contractile properties in all HR lines compared with non-HR mice, and potentially a greater shift than earlier generations of HR mice (Syme et al., 2005), owing to prolonged selection for high levels of voluntary wheel running.

It is unclear why the data presented here show different values of *V*_max_ between muscle complexes from mini- and normal-muscle lines, in addition to differences between normal-muscle HR lines, when Syme et al. (2005) did not find any such differences. It seems unlikely that using the whole triceps surae complex as opposed to individual muscles would lead to this finding – different effects of the mini-muscle phenotype on the various muscles in the complex would be more likely to cancel out than lead to difference between groups. It is possible that these effects are also a consequence of differences in the curve-fitting procedure, or potentially continued responses to ongoing selective breeding over tens of generations.

Syme et al. (2005) also demonstrated a greater curvature in the medial gastrocnemius muscles of mini-muscle groups compared with normal-muscle groups. The curvature of the force–velocity relationship varies from linear to double-hyperbolic, with the reasons for these differences being poorly understood (Alcazar et al., 2019). Hence, it is conceivable than the linearity of the curves measured in this study is a consequence of measuring only the summed output of multiple muscles with different fiber type compositions and morphologies.

Any differences in *V*_max_ and curvature of the force–velocity relationship that are not simply a consequence of curve-fitting procedures, or the measurement of the properties of the entire muscle complex, are likely a reflection of changes in muscle fiber type composition (Schiaffino and Reggiani, 2011). The only study to date that has investigated differences in myosin isoform composition amongst HR lines was at generation 46, and compared soleus, plantaris and gastrocnemius muscles in L3 mini, L7 and L8 mice (McGillivray et al., 2009). That study reported that the soleus had a slightly higher proportion of faster myosin isoforms in the L3 mini-muscle mice, whereas the gastrocnemius and plantaris muscles had a marked reduction in faster myosin isoforms (McGillivray et al., 2009). The large losses of faster fiber types in the mini-muscle lines are consistent with the lower *V*_max_ values reported in these groups here. However, the lack of difference in myosin isoforms between L7 and L8 at generation 46 (McGillivray et al., 2009) are not consistent with the difference in *V*_max_ between these lines reported here (Fig. 3). This may reflect subtle changes not detectable by myosin isoform analysis, or ongoing changes in these groups since generation 46.

### Endurance properties

The soleus and medial gastrocnemius muscles in mice generally fatigue within the first 100 tetanic contractions (Brooks et al., 2018; Cabelka et al., 2019) or within 100–500 s (e.g. see Pagala et al., 1998; Zhao et al., 2005), with the soleus generally being more fatigue-resistant. Such differences in muscle fatigue are, at least in part, attributed to muscle fiber type composition, with Type I fiber abundance being positively correlated with fatigue resistance (see references in Garland, 1988; Schiaffino and Reggiani, 2011). The first study examining endurance properties in muscles from HR mice was at generation 10, and although voluntary exercise on wheels for 2 months improved muscle fatigue resistance, no significant differences were found between HR and control mice (mini-muscle individuals were not present in the sample) (Zhan et al., 1999). Subsequently, Syme et al. (2005) reported that the medial gastrocnemius muscle in mini-muscle individuals had significantly slower rates of fatigue for both isometric force and cyclic net work.

In the present study, we determined endurance *in situ*, in the presence of a functioning circulatory system, across five groups of HR mice. Endurance, measured as the slope of the decline in force over the first 90 tetanic contractions (Endur_0–90_), varied significantly in the triceps surae muscle complex, being shallowest in the mini-muscle mice (L3 mini=−0.00348, L6 mini=−0.00238), steepest in lines L6 and L8 (−0.01676 and −0.01853), and intermediate in L7 (−0.01145) (Fig. 4F). *F*_sustained_/*F*_0_ (sustained isometric force normalized to peak tetanic force) was higher in mini-muscle mice (Fig. 4G), likely because of the higher prevalence of fatigue-resistant muscle fibers (McGillivray et al., 2009; Talmadge et al., 2014). Although the mini-muscle phenotype has drastic effects on muscular endurance, L7 mice also have evolved to have greater endurance as compared with the other normal-muscled HR lines (Fig. 4). As with changes to the force–velocity properties of this line, this was not reflected in fiber type composition at generation 46, and likely represents either subtle changes that could not be detected using the study of myosin isoforms or subsequent changes since generation 46.

### Trade-offs and experimental studies

Despite the clear rationale for, and evolutionary importance of, organismal-level speed–endurance trade-offs underpinned by muscle-level trade-offs, experimental evidence is inconsistent. On the one hand, trade-offs at the muscle level can sometimes be related to organismal-level performance trade-offs. For example, organismal-level trade-offs in the ‘roll-snap’ behavior (the rapid snapping of their wings together above their back) of bearded manakins can be partly explained by contraction–relaxation kinetics in the skeletal muscle that actuates the display (Miles et al., 2018). On the other hand, trade-offs at the level of subordinate traits, such as muscles, can be at odds with speed and endurance metrics at the organismal level. For example, at the organismal level, one study reported an absence of a trade-off between burst swimming performance and endurance capacity in African clawed frogs (Wilson et al., 2002), and another found only marginal evidence for a trade-off between burst (speed and acceleration) and sustained locomotion in lacertid lizards (Vanhooydonck et al., 2014). At the muscle level, studies of these same specimens have revealed highly significant trade-offs between muscular power output and fatigue resistance (Wilson et al., 2002; Vanhooydonck et al., 2014). Selection experiments, in which conditions are tightly controlled, may help to resolve the extent to which these trade-offs could exist and be evolutionarily important.

### Experimental evolution and trade-offs in HR mice

Selection experiments and experimental evolution can be used to study evolution in real time by determining the sequence of phenotypic and behavioral changes that occur during adaptation to a defined selective regime (Garland, 2003; Garland and Rose, 2009; Marchini et al., 2014; Biesiadecki et al., 2020). For example, functional trade-offs involving both muscle and bone underlie trade-offs between running and fighting ability that emerged as greyhounds and pit bulls were developed by artificial selection (Pasi and Carrier, 2003; Kemp et al., 2005). However, few studies have used these approaches to elucidate mechanisms that underlie trade-offs, or examine discrepancies between trade-offs at the organismal level and those found among lower-level traits.

A significant negative correlation between average running speed and time spent running on wheels among the four replicate HR lines was reported at generation 43 (Garland et al., 2011). L3 mini-muscle mice (mini-muscle status was unknown for L6) ran for the fewest minutes per day on wheels, but at the highest average speeds. Mice from L8 ran for the longest durations, but at the slowest average speeds. L7 mice were intermediate for both speed and duration of wheel running.

The muscle complex data presented here for the HR lines from this selection experiment demonstrate a trade-off between muscle speed and endurance across groups (Table 3, Fig. 5). However, this muscle-level trade-off is the opposite of that seen at the organismal level (Garland et al., 2011). Mini-muscle mice (L3 mini and L6 mini) had the highest endurance (Endur_0-90_, *F*_sustained_/*F*_0_) but slowest muscle complexes (*V*_norm_), L6 and L8 had the lowest endurance but fastest muscles, and L7 was intermediate. Hence, although both muscle- (Fig. 5) and organismal-level (Garland et al., 2011) trade-offs between speed and endurance have been observed across HR lines, the former may not underpin the latter.

Muscle- and organismal-level trade-offs might not reflect one another for various reasons. The apparent reversal of the ordering in muscle and organismal level trade-offs (Fig. 5; Garland et al., 2011) may not actually be as much of a discrepancy as it initially appears. Maximal running speeds on wheels (Roach et al., 2012) are well below maximal sprint speeds (Dohm et al., 1996; Girard et al., 2001; Dlugosz et al., 2009; Claghorn et al., 2017), and maximal sprint speed is reduced in L3 mini individuals as compared with L7 and L8 individuals (Dlugosz et al., 2009). Hence, if we had measured sprint speed (Dlugosz et al., 2009) and running endurance (Meek et al., 2009) as metrics of organismal-level speed and endurance in this generation, we may not have found any evidence of a trade-off. This would be more in line with previous studies that show evidence of muscle-level trade-off, but no, or marginal, trade-offs at the organismal level (Wilson et al., 2002; Vanhooydonck et al., 2014). However, the potential to draw different conclusions regarding trade-offs at submaximal and maximal activity levels highlights the complexity of trade-off studies (for a general review of this, see Garland et al., 2022), and calls into question the relationship between muscle properties and organismal performance during submaximal tasks.

Hence, there is potential for there not to be a complete reversal of muscle- and organismal-level trade-offs if different organismal-level metrics were used. However, it does still seem likely that there is some degree of discrepancy. An obvious potential cause of differences between muscle- and organismal-level traits is that muscle properties are only one of many lower-level traits that contribute to whole-animal locomotor abilities. Although metrics of maximal sprint speed are relatively closely related to aspects of muscle properties among human athletes (e.g. see Komi, 1984), other morphological, neural and biomechanical traits are also important. And measures of endurance encompass many additional lower-level traits besides muscle physiology, including biomechanics, oxygen transport and delivery, thermoregulatory abilities and additional cellular biochemical processes (discussed in Garland, 1988; Jones and Lindstedt, 1993; Schiaffino and Reggiani, 2011; Vanhooydonck et al., 2014; Thompson, 2017). Higher-level factors, such as differences in motivation, are also likely to have major effects on running speed and duration (e.g. see Rhodes et al., 2005; Claghorn et al., 2016; Garland et al., 2016; Roberts et al., 2017; Saul et al., 2017 and references therein).

Although many of the factors mentioned above are beyond the scope of this study, here we consider in more detail the potential for muscle and biomechanical factors to obscure the effects of muscle-level trade-offs, as a significant amount of literature exists on these topics in the HR mice. Individuals may, at least in part, compensate for the functional constraints that particular muscles impose by activating additional agonistic muscles (discussed in Wilson and James, 2004) and changing their gait. HR mice have evolved narrower stance width than control mice lines, mini-muscle mice have increased duty factor and larger paw contact areas (Claghorn et al., 2017), and female HR mice run more intermittently than control mice (Girard et al., 2001).

One specific example of how the intersection of muscle and biomechanical factors could potentially contribute to discrepancies between muscle- (Fig. 5) and organismal-level (Garland et al., 2011) trade-offs is the reduced capacity of the triceps surae to support body weight in mini-muscle lines (Fig. 2B). Locomotor endurance is determined not only by the fatigue resistance of the muscle fibers, but also by the total force capacity of the muscle relative locomotor demands. The reduced capacity of the triceps surae of mini-muscle mice to support body weight likely reduces the reserve capacity of the muscular system, meaning that any fatigue of the individual fibers will likely have a greater contribution to organismal fatigue. However, this effect may be offset by further biomechanical changes in mini-muscle mice, such as higher duty factors (Claghorn et al., 2017), which reduce peak force demands during running. Hence, although reduced muscle reserve certainly is not a definitive explanation for the discrepancies seen between muscle and organismal levels, it does highlight the emergent nature of organismal performance, and the limited role that any given tissue-level trade-offs may play, particularly during submaximal activities.

## Supplementary Material

10.1242/jexbio.244083_sup1 Supplementary information
