# Supplementary material for: Trade-offs in muscle physiology in selectively bred high runner mice
Source: J Exp Biol. 2022 Dec 9;225(23):jeb244083. doi: 10.1242/jeb.244083 (PMC9789404; doi:10.1242/jeb.244083)
Supplement: Supplementary information [file jexbio-225-244083-s1.pdf]

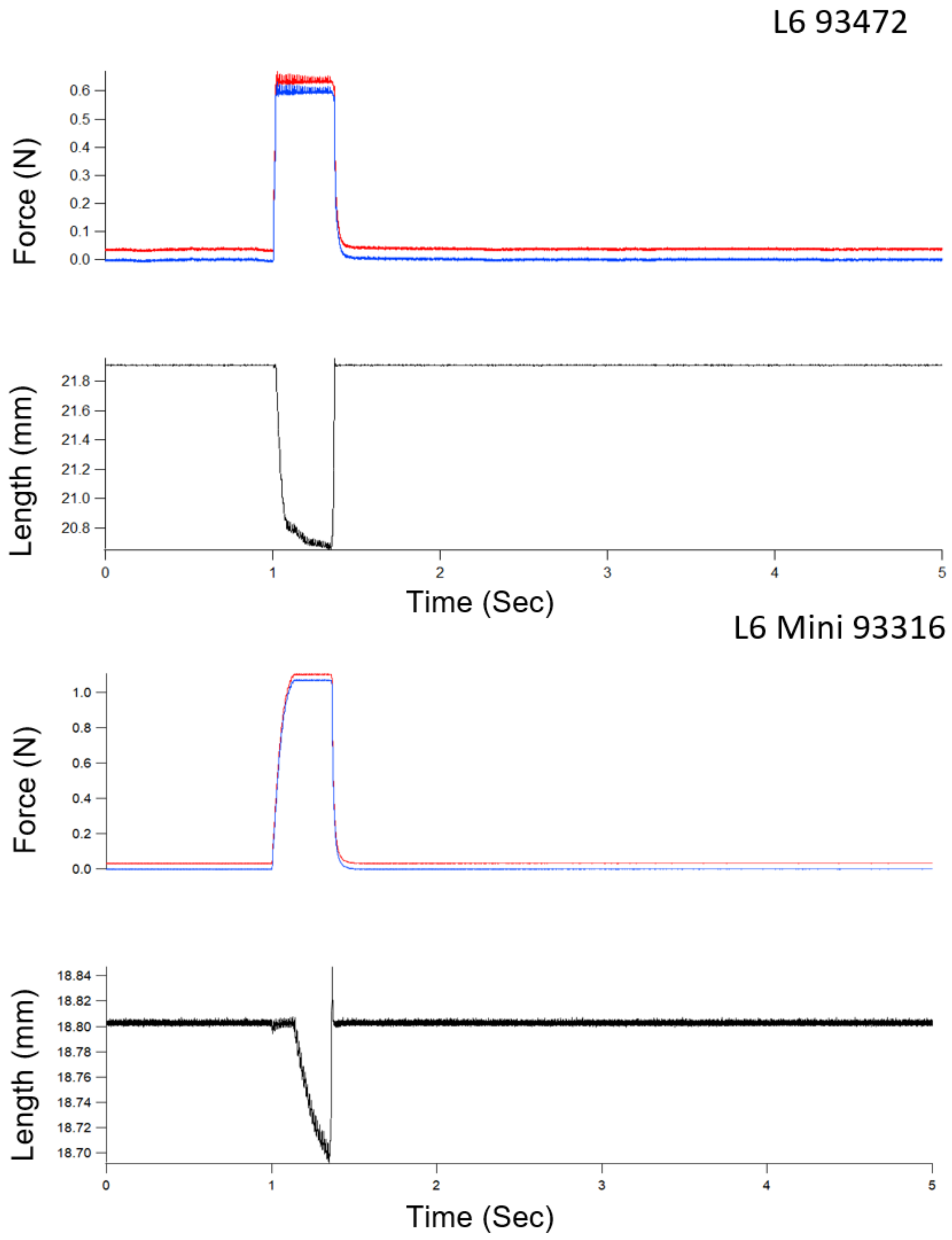

Fig S1. Sample traces of individual isotonic contractions. Peak shortening velocity and force are extracted from these traces, and used to construct force-velocity curves.

Journal of Experimental Biology • Supplementary information

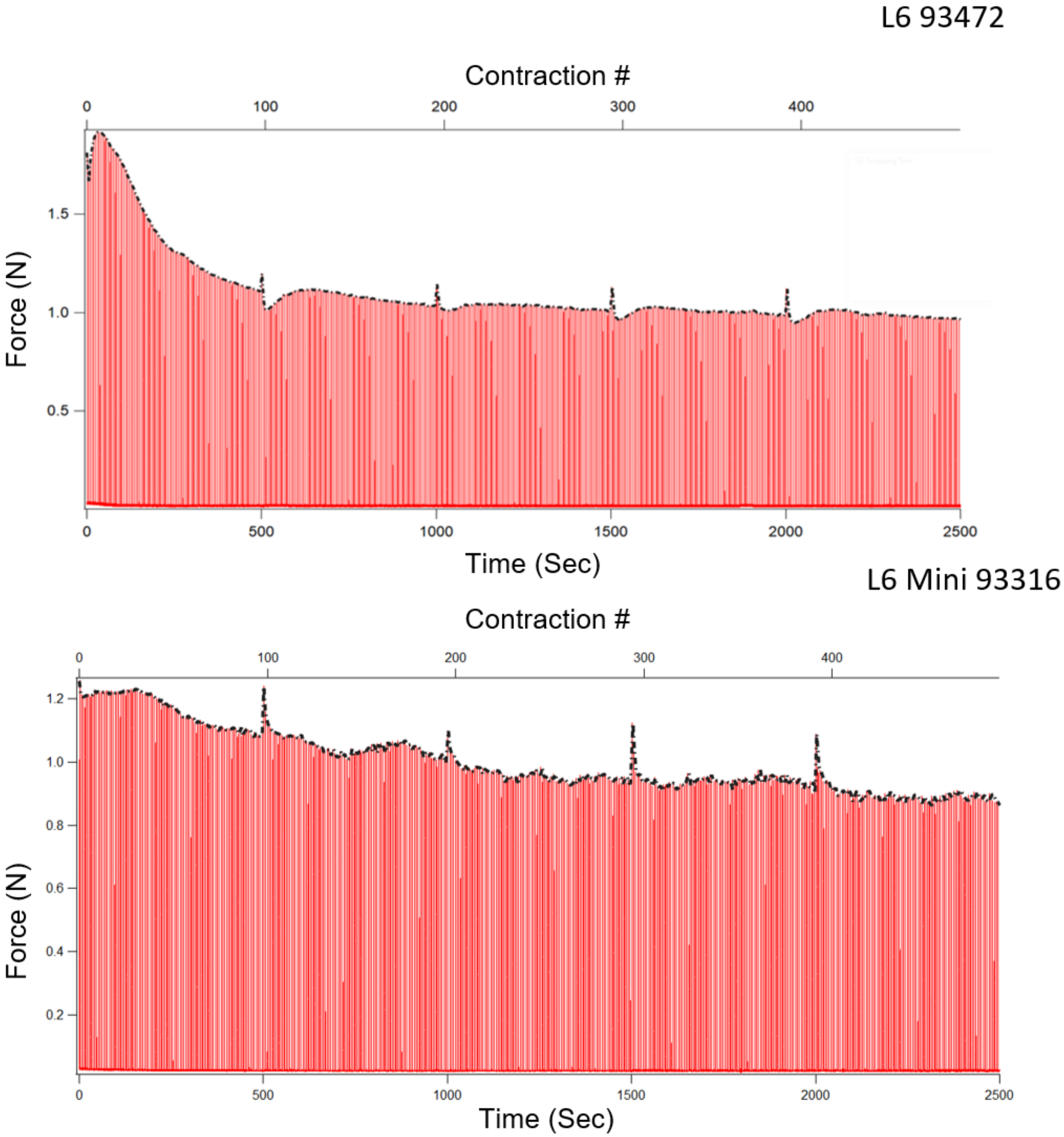

Fig. S2. Sample traces of repeated isometric contractions used to generate the endurance wave profiles from which Endur0-90, Sustained F, and Sustained F/F0 were calculated.

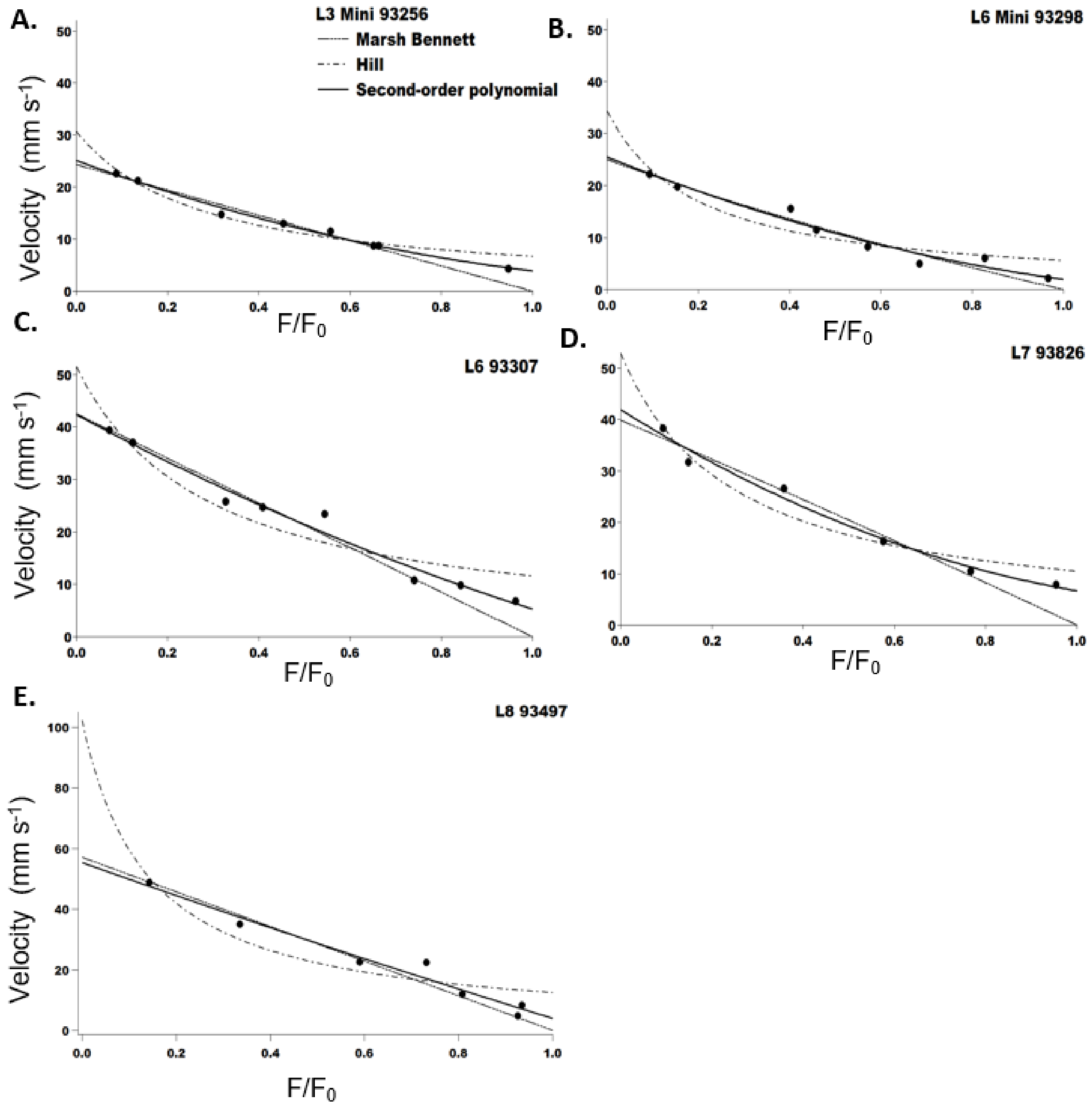

Fig. S3. Representative force-velocity trace for L3 Mini (A), L6 Mini (B), L6 (C), L7 (D), and L8 (E) with (F/F0) on the x-axis and absolute shortening velocity on the y-axis. The force-velocity points were curve-fitted using the Hill equation, Marsh-Bennet equation, and second-order polynomials. Maximal shortening velocity mm s-1 estimates using the three fits are visually rendered.

Journal of Experimental Biology • Supplementary information
